# The Notch Signaling Regulates CD105 Expression, Osteogenic Differentiation and Immunomodulation of Human Umbilical Cord Mesenchymal Stem Cells

**DOI:** 10.1371/journal.pone.0118168

**Published:** 2015-02-18

**Authors:** Tao Na, Jing Liu, Kehua Zhang, Min Ding, Bao-Zhu Yuan

**Affiliations:** 1 Cell Collection and Research Center, National Institutes for Food and Drug Control, Beijing, 100050, China; 2 National Institute for Occupational Safety and Health, Morgantown, West Virginia, 26505, United States of America; The University of Adelaide, AUSTRALIA

## Abstract

Mesenchymal stem cells (MSCs) are a group of multipotent cells with key properties of multi-lineage differentiation, expressing a set of relatively specific surface markers and unique immunomodulatory functions. IDO1, a catabolic enzyme of tryptophan, represents a critical molecule mediating immunomodulatory functions of MSCs. However, the signaling pathways involved in regulating these key properties still remain elusive. To investigate the involvement of Notch signaling as well as other potential signaling pathway(s) in regulating these critical properties of MSCs, we treated human umbilical cord-derived mesenchymal stem cells (hUC-MSCs) with γ-secreatase inhibitor I (GSI-I), which inhibits both Notch signaling and ubiquitin-proteasome activities. It was shown that the GSI-I treatment resulted in apoptosis, reduced expression of surface markers CD73, CD90 and CD105, reduced osteogenic differentiation, and reduction of the hUC-MSCs-mediated suppression of Th1 lymphocyte proliferation and the IFN-γ-induced IDO1 expression. Through distinguishing the effects of GSI-I between Notch inhibition and proteasome inhibition, it was further observed that, whereas both Notch inhibition and proteasome inhibition were attributable to the reduced CD105 expression and osteogenic differentiation, but not to the induced apoptosis. However, Notch inhibition, but not proteasome inhibition, only contributed to the significant effect of GSI-I on Th1 proliferation probably through reducing IDO1 promoter activity. In conclusion, the Notch signaling may represent a very important cell signaling capable of regulating multiple critical properties, especially the immunomodulatory functions of MSCs.

## Introduction

Mesenchymal stem cells (MSCs) represent a group of fibroblast-like multipotent cells with abilities to differentiate into multi-lineage cells, such as chondrocytes, osteocytes, adipocytes, neurons, and hepatocytes. They were identified first in bone marrow, and later in almost all tissues, including adipose tissue, placenta, and umbilical cord [1–5].

MSCs can be minimally defined regardless of tissue origins by 1) adherent growth on plastic surface; 2) expressing a set of relatively specific surface markers, such as positive markers CD73, CD90 and CD105 expressing in over 95% of cell populations, and negative markers CD14, CD34, CD45 and HLA-DR in less than 2% of cell populations; 3) abilities to differentiate into osteocytes, chondrocytes and adipocytes *in vitro* [6].

Even though the positive surface markers have been used for defining MSCs, the expression of them may not always be stable. Differentiation status, special treatments, or certain pathological situations may affect their expressions. For example, adipogenic differentiation, damage repair from bone fracture, or osteogenic differentiation through mechanical stimulation may cause the reduced expression of CD105, CD90 or CD73, respectively [7–9].

In addition to the expression of surface markers and progenitor properties, MSCs of various origins also possess unique immunomodulatory and anti-inflammatory functions, which make them very promising in MSC-based therapies. Currently, there are approximately 400 registered clinical trials worldwide for testing MSC-based products in treating various diseases (http://clinicaltrials.gov/), such as diabetes, multiple sclerosis, cardiovascular diseases, liver fibrosis, etc, underlying which are the abnormal immune responses or uncontrolled inflammatory responses [10].

The immunomodulatory functions of MSCs are represented in part by their abilities to inhibit proliferation of pro-inflammatory immune cells, such as the Th1 subset of CD4^+^ lymphocytes, but promote maturation of Regulatory T lymphocytes (Tregs) [11]. Such functions are mediated by a number of active molecules, such as TGF-β, HGF, PGE2, IL-10, and IDO1 [12], among which, IDO1, or indolamine 2,3-dioxygenase 1, has become a recent focus of the immunomodulation studies of MSCs. IDO1 needs to be activated first for its expression by inflammatory cytokines, such as IFN-γ and TNF-α, and then exerts its immunomodulatory activities through breaking down tryptophan into kynurenine and other downstream metabolites along the kynurenine pathway [13–15].

The afore-mentioned properties are associated with the key quality attributes of the MSC-based products [16]. However, the relationship among the quality attributes still remains unclear. Among all approaches for uncovering the possible relationship, identifying key signaling pathways involved in regulation of the critical properties is believed to be an effective one. A number of cell signaling pathways, such as TGF-β, Wnt, MAPK, and Notch pathways, have been reported involving in regulating fate, viability or differentiation of stem cells [17]. Among them, the Notch signaling may serve as a more versatile signaling capable of regulating multiple functions of various stem cells. For example, the Notch signaling determines fates of embryonic stem cells, affects viability of cancer stem cells or their sensitivity to chemo- or radio-therapies, and coordinates osteogenic differentiation of MSCs [18,19]. It may also be involved in regulating immune system. Recent data has shown that the Notch signaling was involved in production of IDO1 in dendritic cells [20] and in MSCs-mediated increase of Tregs [21].

The Notch proteins are a family of transmembrane receptor proteins, the pivotal components of the Notch signaling pathways. In mammals, there are four Notch (Notch1–4) receptor proteins and five Notch ligands, which are Delta-like1, 3, and 4 and Jagged 1 and 2. Activation of Notch signaling is initiated by binding of Notch receptor with its ligand between adjacent cells, as followed by two successive proteolytic cleavages, which are mediated sequentially by TNF-converting enzyme and γ-secretase/presenilin complex [22]. The cleavages result in release of intracellular domain of Notch (NICD) from plasma membrane and translocation of NICD into the nucleus, where NICD binds the CSL protein complex and turns the complex from a transcriptional repressor into a transcriptional activator with consequent activation of downstream effector proteins, such as Hes and Hey proteins [23].

As the γ-secretase-mediated cleavage is the rate-limiting step of initiating Notch signaling, γ-secretase inhibitors (GSIs) have been frequently used as the effective research tools in uncovering novel functions of the Notch signaling [24]. Among the most commonly used GSIs was a leupeptin analogue, Z-Leu-Leu-Nle-al, also called GSI-I, which is structurally similar to proteasome inhibitor Bortezomib[25,26].

To determine the involvement of Notch signaling in regulating the quality-associated properties of MSCs, we initially employed GSI-I to treat human umbilical cord-derived MSCs (hUC-MSCs), then analyzed the consequences of the treatment, as followed by distinguishing Notch signaling inhibition and proteasome inhibition of GSI-I in hUC-MSCs. Through this approach, we revealed that the Notch signaling is involved in regulating surface markers, CD105 in particular, osteogenic differentiation, and immunomodulation of hUC-MSCs, thus revealing that the relationship among the key quality-associated properties lies in the Notch signaling, which may be the key signaling for maintaining the integrity of hUC-MSCs. More interestingly, it was revealed that the Notch-regulated immunomodulation was likely achieved through promoting IDO1 transcription.

## Materials and Methods

### 1. Materials

(1) Chemicals: GSI-I (Z-Leu-Leu-Nle-al), a γ-secretase inhibitor, and 1-L-MT (1-Methyl Tryptophan, an IDO1 inhibitor, were purchased from Sigma Aldrich (St. Louis, MI，USA); Bortezomib, a proteasome inhibitor, was from ChemieTek (Indianapolis, IN, USA). (2) Antibodies: the antibodies against caspases 3 and 7, PARP, phospho-histone H2AX (at Ser139), Akt, phospho-Akt (at Ser473), Stat3, phospho-Stat3 (at Tyr705), Survivin, IDO1, HDAC1 and HDAC2 were from Cell Signaling (Danvers, MA, USA); the antibodies against Mcl-1, Hes1, NFkB, cyclin A and D were from Santa Cruz Biotechnology (Santa Cruz, CA, USA); the antibodies against ubiquitin, p21 and actin were from Sigma Aldrich. All antibodies conjugated with different fluorescent dyes used in flow cytometry assay were from BD (Franklin Lakes, NJ, USA). (3) Cells: hUC-MSCs with the catalog number of 20120822C6P5 were gifted anonymously from TuoHua Biotech company (Siping, China), where the cells were isolated and purified from Wharton’s Jelly of a discarded umbilical cord of a normal laborby following the previously described procedures [27]. The major MSC properties, such as expression of surface markers and differentiation potentials to osteocytes, chondrocytes and adipocytes, and microbiological safety were validated in our laboratory. Peripheral blood mononuclear cells (PBMCs) were freshly prepared using a conventional Ficoll method [28] from whole blood of healthy donors provided anonymously from Beijing Red Cross Center (37# of North 3^rd^ Ring Road, Beijing) with consent for use in research. All data analysis associated with the use of hUC-MSCs and PBMCs in this study was conducted anonymously.

### 2. Detection of cell surface markers

The multi-color flow cytometry using BD Stemflow hMSC Analysis Kit was employed to detect expression of surface markers. A previous report was followed in cell staining and data analysis [29].

### 3. Osteogenic differentiation

The osteogenic differentiation was used in this study to represent differentiation potentials of hUC-MSCs. The procedures reported in a previous study were followed in induction of osteogenic differentiation, cell staining and data analysis [29].

### 4. Semi-quantitative RT-PCR

A semi-quantitative RT-PCR was employed to detect transcriptional changes of RGC32 and IDO1 in hUC-MSCs following GSI-I treatment [30]. Briefly, 1 μg of total RNA isolated using Trizol agent from the cells after each treatment were reverse-transcribed using SuperScript III First-Strand Synthesis System (Invitrogen), and then amplified by PCR using each specific primer set. The expression of GAPDH gene was used as an internal control in the semi-quantitation. The primers used in this study were: IDO1, AAGTGGGCTTTGCTCTGC and GGCAAGACCTTACGGACA; RGC32, GCCACTTCCACTACGAGGAG and GTGGCCTGGTAGAAGGTTGA; GAPDH, ACCACAGTCCATGCCATCAC and TCCACCACCCTGTTGCTGT.

### 5. Inhibition of Th1 lymphocyte proliferation by hUC-MSCs

Fresh PBMCs were co-cultured with hUC-MSCs in 1:5 ratio for 24 h in RPMI 1640 complete medium containing 10% FBS and then stimulated with 25 ng/mL PHA, 1 μg/mL Ionomycin and 10 μg/mL BFA for another 5 hours. Then, the CD8^─^/INF-γ^+^ cells were gated in a flowcytometer (FACSCalibur, BD) with the relevant antibodies to determine the amount of Th1 lymphocytes.

### 6. Western blotting

The procedures of conventional Western blotting were followed to monitor changes in expression of relevant proteins in hUC-MSCs following various treatments. Briefly, cell lysates were prepared using RIPA buffer containing proteinase inhibitor cocktail (Sigma, Milwaukee, WI), separated in 10–14% PAGE gel and transferred onto nitrocellulose membrane. The signals were detected using ECL Advance Western Blotting Detection Kit (GE Healthcare, Piscataway, NJ).

### 7. Immunoprecipitation (IP) for detecting the poly-ubiquitinated Mcl-1 protein

Cell lysates extracted using RIPA buffer from the cells treated with GSI-I or Bortezomib were incubated with1 μg Mcl-1 antibody at 4°C overnight, then incubated with 300 μl protein A/G agarose at 4°C for 1 h. After washing three times at room temperature, the agarose-bound cell lystates were then analyzed by Western blotting using ubiquitin antibody.

### 8. siRNA transfection

The procedures described previously for siRNA transfection were followed to transfect Notch1 siRNA or control siRNA into hUC-MSCs [31] and the silencing of the target gene was determined by Western blotting. Each siRNA silencing experiment was repeated at least twice. The siRNA sequence for Notch1 is 5′-CACCAGUUUGAAUGGUCAAtt-3′ [32]. The randomly scrambled siRNA was used as negative control.

### 9. Cell viability assay

Ten microliter of WST-8 agent (Dojindo, Kumamoto, Japan) was directly added into each well of a 96-well plate with each well containing 1–2 × 10^4^ test cells. Following the incubation at room temperature for 4 h, the cell viability in each well was determined by measuring absorbance at 450 nm using a microplate reader (Molecular Devices, Sunnyvale, CA).

### 10. Luciferase assay for detecting IDO1 promoter activity

The pIDO1-Luc vector was constructed by cloning the IDO1 promoter covering a region of 1576 bp upstream of the ATG starting codon of the gene into the pGL3-Basic vector [33]. For testing IDO1 promoter activity, the freshly plated hUC-MSCs in approximately 90% confluence in 24-well plates were transfected with pIDO-Luc. After transfection for 24 h, the cells were treated with 2 ng/mL IFN-γ. At 24 h after treatment, the luciferase activity was measured using Glomax Luminometer and Bright-Glo Luciferase Assay System (Promega). Each sample was tested in triplicates and each test was repeated three times. The results were expressed as relative luciferase units (RLU) after adjustment by pGL3-Basic transfection, which was used as negative control.

### 11. Data analysis

The Data from cell viability, detection of surface markers, luciferase assay and Th1 lymphocyte proliferation were expressed as means±SEM of at least three separate experiments. Comparison between group means was assessed using one-way analysis of variance with Newman—Keuls posttest using GraphPad Prism 4.0 Software (San Diego, CA). The difference with *p*<0.05 was considered statistically significant.

## Results

### 1. The GSI-I treatment induced a caspase-dependent apoptosis in hUC-MSCs accompanied by activation of anti-apoptosis mechanisms

**Fig 1:**
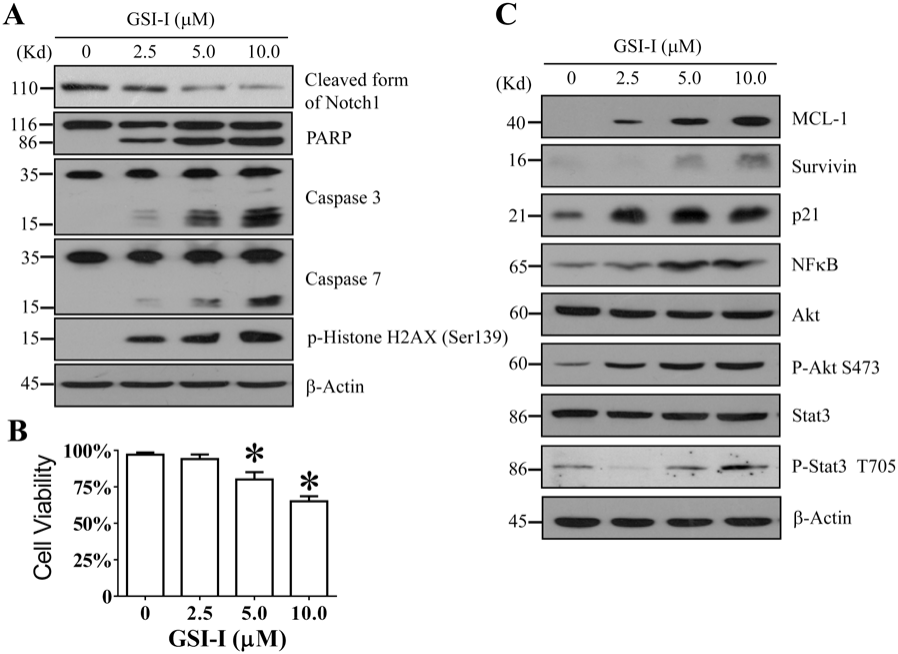
The GSI-I treatment induces a caspase-dependent apoptosis in hUC-MSCs at 24 h after treatment. A. Western blotting shows that the treatment reduces the cleaved Notch1, induces cleavage of caspases 3 and 7, PARP proteins, and increases p-Histone H2AX. B. WST-8 cell viability assay shows that the treatment significantly reduces viability of hUC-MSCs. The * indicates statistical significance with *p*<0.05. C. Western blotting shows that the treatment elevates Mcl-1, p21, Survivin, NFkB, p-Akt and p-Stat3.

In this study, hUC-MSCs were used as the representative of human MSCs. To reveal the activities of Notch signaling in hUC-MSCs, we treated 1×10^6^ cells with 2.5–10 μM GSI-I for 24h, then analyzed the consequences of the treatment. It was first observed in Western blotting that the treatment caused a significant dose-dependent reduction of the cleaved Notch1, the active form of Notch1 protein (Fig. 1A), suggesting that the GSI-I treatment significantly reduced the activity of Notch signaling. The Notch1 protein was tested because it was the major form of the Notch family proteins expressing in hUC-MSCs (data not shown). It was further observed that greater than 5 μM GSI-I also caused a significant reduction of cell viability accompanied by dose-dependent protein cleavages for caspases 3 and 7, PARP, as well as an increase of phosphorylated Histone H2AX (Ser139), an indicator of DNA damage, suggesting that the GSI-I treatment induced a caspase-dependent apoptosis in hUC-MSCs (Fig. 1A & 1B) [34]. Meanwhile, a significant dose-dependent elevation was observed for Mcl-1, p21, Survivin, phospho-Akt and phospho-STAT3 proteins, which represent different anti-apoptotic mechanisms, suggesting that the negative feedback mechanisms for protecting the cells from the GSI-I-induced apoptosis was also activated in hUC-MSCs (Fig. 1C).

### 2. GSI-I reduced expression of positive MSC surface markers

**Fig 2:**
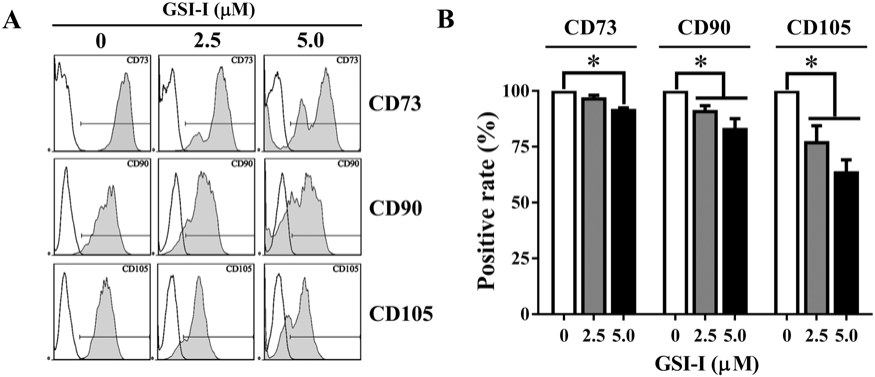
The GSI-I treatment reduces expression of positive MSC surface markers. Representative histogram from a flow cytometry assay shows that, while over 95% untreated hUC-MSCs are positive for CD73, CD90 and CD105 (A), the treatment significantly reduces the positivity for each surface marker with the most significance seen for CD105 reduction. The * indicates the statistical significance with *p*<0.05 (B).

Following the observation of apoptosis, we then attempted to determine whether the GSI-I treatment could also cause any change in MSC surface markers. It was found via flow cytometry that, while over 95% of untreated hUC-MSCs expressed CD73, CD90 and CD105, the treatment with greater than 5 μM GSI-I for 24 h caused an obvious reduction of all these markers with the most significant reduction seen in CD105 (Fig. 2).

### 3. GSI-I reduced osteogenic differentiation of hUC-MSCs

**Fig 3:**
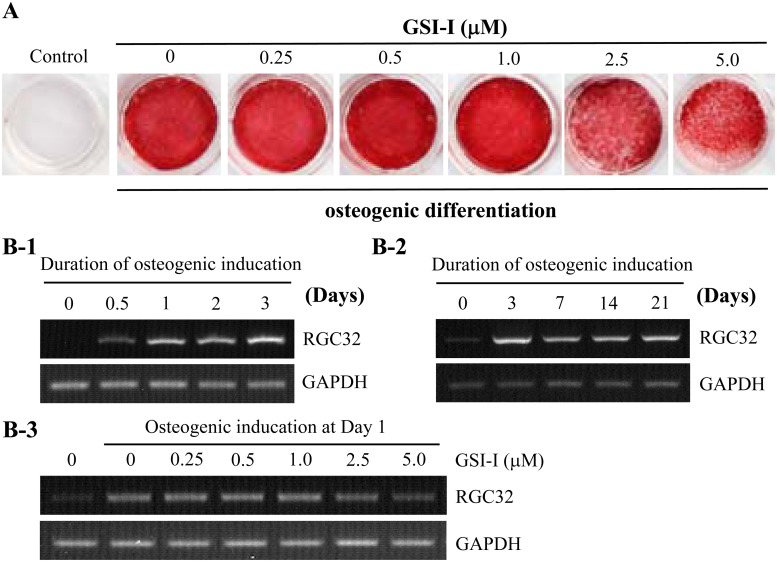
The GSI-I treatment reduces osteogenesis of hUC-MSCs. A. The treatment inhibits intracellular calcium deposition as detected by Alizarin Red staining at 14 d after osteogenic induction. B. The semi-quantitative RT-PCR shows that the osteogenic induction induces RGC32 gene expression starting at 12 h and reaching to a plateau at day 3 (B-1, B-2). However, the GSI-I treatment with greater than 2.5 μM GSI-I significantly reduces RGC32 transcription at 24 h after treatment (B-3). The cells without osteogenic induction serve as negative control.

To determine the effects of GSI-I on differentiation potential of hUC-MSCs, we employed osteogenic differentiation as a representative, in which a standard ascorbic acid-based protocol was used to induce osteogenic differentiation followed by Alizarin Red staining to detect intracellular calcium deposition [35]. In addition, a semi-quantitative PCR was also used to test expression of RGC32 gene, which is up-regulated during osteogenic differentiation of MSCs [30]. It was found that significant osteogenic differentiation was observed as indicated by a strong Alizarin Red staining at day 21, or a persistently increased expression of RGC32 from day 1 to day 21 after the induction. However, the co-treatment with greater than 2.5 μM GSI-I significantly reduced RGC32 transcription at day 1 and Alizarin Red staining at day 21 (Fig. 3A & B), thus demonstrating that the GSI-I treatment reduced osteogenic differentiation potential of hUC-MSCs.

### 4. GSI-I inhibited hUC-MSCs-mediated suppression of Th1 lymphocyte proliferation and reduced IFN-γ-induced IDO1 expression

**Fig 4:**
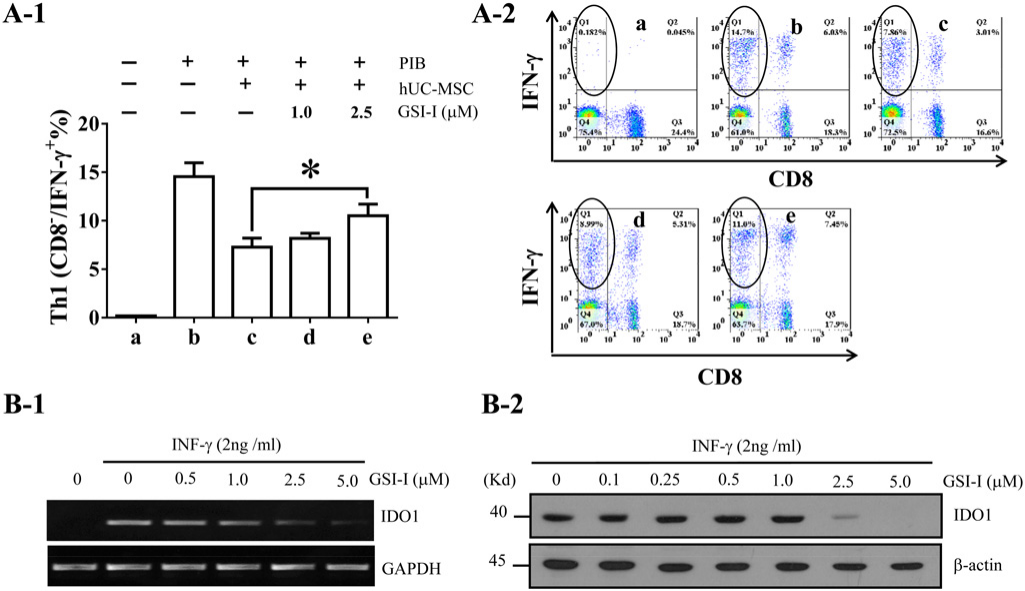
The GSI-I treatment inhibits hUC-MSC activities in suppressing Th1 lymphocyte proliferation and IDO1 expression. A. Flow cytometry assay shows that co-culturing of hUC-MSC with PBMC in 1:5 ratio significantly inhibits Th1 lymphocyte proliferation. However, the treatment with 2.5 μM GSI-I significantly inhibits the effect of hUC-MSCs on Th1 proliferation. A-1: bar figure; A-2: original spectrogram of a representative Th1 lymphocyte analysis. Th1 lymphocytes are CD8-/IFN-g+ cells as circled in the spectrogram. B. The GSI-I treatment reduces the IFN-γ-induced IDO1 expression. Both semi-quantitative RT-PCR (B-1) and Western blotting (B-2) show that, while 2ng/mL IFN- γ induces IDO1 expression in hUC-MSCs, greater than 2.5 M GSI-I dramatically reduces the IDO1 expression.

It has been well established that MSCs can inhibit proliferation of the Th1 subset of CD4^+^ lymphocytes, and such activity is mediated at least in part by IDO1 upon induction by pro-inflammatory cytokines [36]. To determine the effects of GSI-I on Th1 proliferation, we co-cultured hUC-MSCs with PBMC in the presence of GSI-I for 24 h and then incubated with 25 ng/mL PHA, 1 μg/mL Ionomycin and 10 g/mL BFA (PIB) for 5 h. It was found via flow cytometry that, while 1×10^5^ hUC-MSC reduced approximately 50% of Th1 proliferation, the GSI-I treatment significantly blocked this effect (Fig. 4A). In a separate experiment, direct treatment on PBMC with 2.5 μM GSI-I alone slightly reduce the Th1 proliferation suggesting that GSI-I alone did not contribute to the effect of the GSI-I-treated hUC-MSCs (data not shown).

Next, we tested IDO1 expression in hUC-MSCs following co-treatment with GSI-I and IFN- γ. It was found that, while 2 ng/mL IFN- γ induced a significant expression of IDO1, greater than 2.5 μM GSI-I dramatically reduced this activity, as detected by both Western Blotting and RT-PCR (Fig. 4B-1 & B-2). In a separate experiment, it was found through Western blotting that the GSI-I treatment alone on PBMC with or without lymphocyte stimulation did not elicit any detectable IDO1 protein expression (data not shown) in PBMC. All data thus suggested that the GSI-I treatment inhibited immunosuppressive properties of hUC-MSCs probably through inhibiting IDO1 expression.

### 5. GSI-I inhibited both Notch signaling and proteasome activities

**Fig 5:**
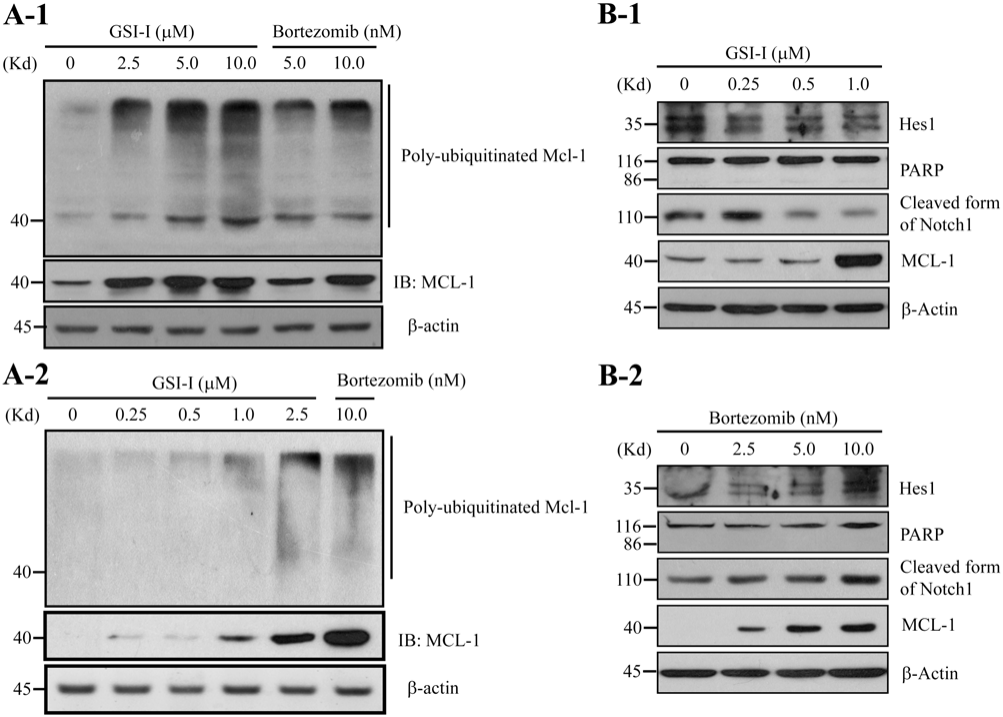
The GSI-I treatment inhibits both Notch and ubiquitin-proteasome signaling in hUC-MSCs. A. Both GSI-I and Bortezomib inhibit ubiquitin-proteasome signaling. The Western blotting following immunoprecipitation shows that the treatment with 1.0–10.0 μM GSI-I or 5–10 nM Bortezomib significantly elevates the level of ubiquitin-conjugated Mcl-1 protein. A-1 is from the data using 2.5 μM-10.0 μM of GSI-I and 2.5–5 nM Bortezomib and A-2 is from the data using 0–2.5 μM of GSI-I and 10 nM of Bortezomib. B. GSI-I, but not Bortezomib, inhibits the Notch signaling. The Western blotting shows that, whereas 0.25–1 μM GSI-I reduce levels of the cleaved form of Notch1 and Hes1 proteins (B-1), 2.5–10 nM Bortezomib however significantly increase Hes1 protein level (B-2).

Given that GSI-I shares a structural similarity with proteasome inhibitor Bortezomib and induced elevation of Mcl-1 and Survivin protein, which are well-established degradation targets of the proteasome [25,34], it was likely that the effects of GSI-I on hUC-MSCs might also be attributable to proteasome inhibition. To test this hypothesis, we employed an IP assay to examine the level of polyubiquitin-conjugated Mcl-1 protein in GSI-I-treated cells. It was found that the treatment with greater than 2.5 μM of GSI-I resulted in a significant increase in polyubiquitin-conjugated Mcl-1 protein (Fig. 5A-1), thus supporting that the GSI-I treatment inhibited both Notch signaling and proteasome activities in hUC-MSCs.

### 6. Both Notch1 inhibition and proteasome inhibition were involved in the GSI-I-induced reduction of surface markers, but not apoptosis

To determine the contribution of Notch inhibition or proteasome inhibition, or the combination of both events, to the GSI-I-induced apoptosis and reduction of surface markers, we then treated the cells with low-dose GSI-I, i.e. 0.25 to 1.0 μM, or proteasome inhibitor Bortezomib, assuming that the effects of low-dose GSI-I may exert only Notch inhibition whereas high-dose GSI-I may exhibit both Notch inhibition and proteasome inhibition. It was found that, whereas low-dose GSI-I reduced both Notch1 cleavage and Hes1 protein level (Fig. 5B-1), 2.5–10 nM Bortezomib significantly increased both Hes1 and Mcl-1 protein levels (Fig. 5B-2). Consistently, we also found that GSI-I of greater than 0.5 μM of GSI-I and 5–10 nM Bortezomib, but not 0.25–0.5 μM GSI-I, increased the level of polyubiquitin-conjugated Mcl-1 protein, thus supporting that the low-dose GSI-I were only Notch-inhibitory, but high-dose GSI-I were both Notch-inhibitory and proteasome-inhibitory (Fig. 5A-1 & 5A-2). Therefore, elevation of Mcl-1 and Hes1 induced by high-dose GSI-I was mainly the consequence of proteasome inhibition. Furthermore, it was found that either Bortezomib or low-dose GSI-I caused no apoptosis as neither PARP cleavage (Fig. 5B-1 & 5B-2) nor reduced cell viability (data not shown) were observed.

**Fig 6:**
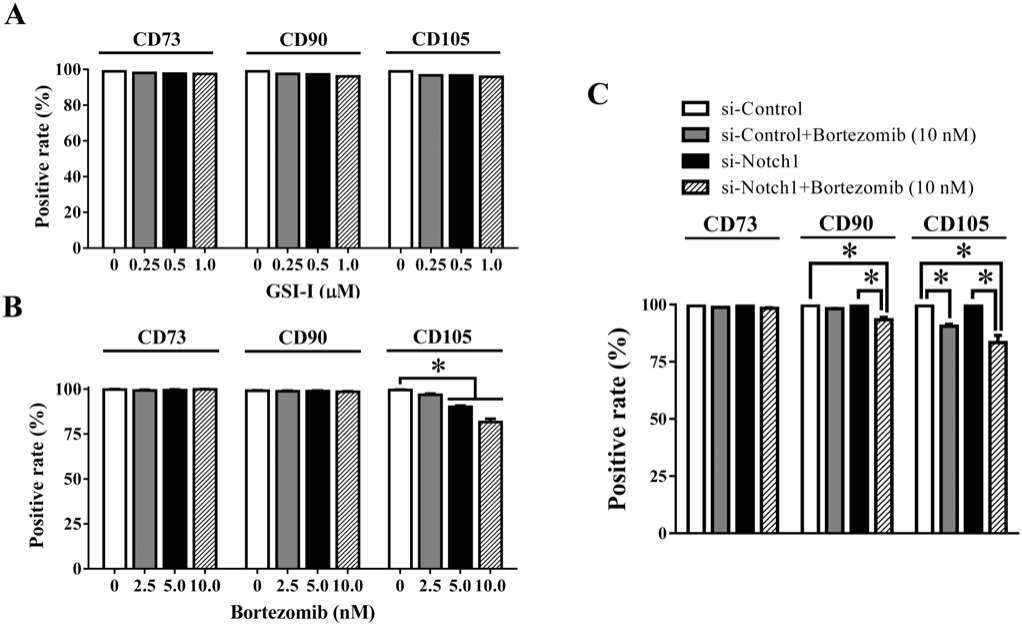
Both Notch signaling and proteasome pathway are involved in regulating CD105 expression in hUC-MSCs. Flow cytometry assay shows that 0.25–1 μM GSI-I do not induce changes in expression of CD73, CD90 and CD105 (A), but 5–10nM Bortezomib significantly inhibits the expression of CD105, but not CD73 and CD90 (B); Moreover, while the siNotch1 transfection alone has no effect on expression of surface markers, it further increases the inhibitory effect of 10 nM Bortezomib on CD105, slightly on CD90, but not on CD73 (C). The * indicates the statistical significance with *p*<0.05.

Next, it was found that low-dose GSI-I did not induce any change of surface markers (Fig. 6A), but Bortezomib caused a clear reduction of CD105, but not CD73 and CD90 (Fig. 6B), suggesting that the apoptosis induced by high-dose GSI-I was not the consequence of either Notch inhibition or proteasome inhibition, but proteasome inhibition may be involved in the reduction of cell surface markers, the CD105 reduction in particular.

**Fig 7:**
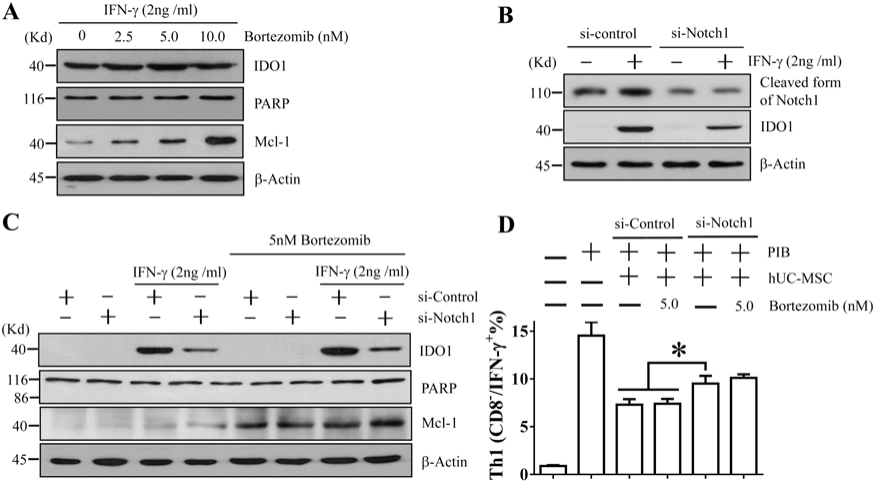
The Notch signaling plays a predominant role in maintaining immunomodulatory activities of hUC-MSCs. A. Western blotting shows that Bortezomib alone does not affect the IFN-γ-induced IDO1 expression, does not induce apoptosis as no PARP cleavage was induced, but elevates Mcl-1 protein level. B. Western blotting shows that siNotch1 transfection can silence approximately 50% of IDO1 protein expression, but does not induce apoptosis. C. Western blotting shows that treatment with 5 nM Bortezomib does not further enhance the effect of siNotch1 transfection on IDO1 reduction. In addition, Bortezomib and siNotch1 together do not induce apoptosis as no PARP cleavage is observed. D. The flow cytometry assay shows that siNotch1 transfection alone can significantly reduce the inhibitory effect of hUC-MSCs on Th1 lymphocyte proliferation. Bortezomib alone shows no such effect and does not enhance the effect of siNotch1 either. The * indicates statistical significance with *p*<0.05.

To further determine the causes of GSI-I-induced apoptosis and reduction of surface markers, we then transfected hUC-MSCs with Notch1 siRNA (or siNotch1) together with Bortezomib treatment. The siNotch1 transfection reduced approximately 50% expression of Notch1 protein (Fig. 7B). However, siNotch1 alone or in combination with Bortezomib induced no apoptosis, as no cell death (data not shown) and no PARP cleavage were observed (Fig. 7C). The effect of Bortezomib was confirmed by the elevated Mcl-1 protein level (Figs. 5B-2, 7A & 7C). In addition, while siNotch1 alone caused no change in expression of all surface markers, it clearly enhanced the effect of Bortezomib on the reduction of CD105 (Fig. 6C), suggesting that both Notch1 inhibition and proteasome inhibition contributed to the reduction of CD105, but not to the apoptosis.

### 7. Both Notch inhibition and proteasome inhibition were attributable to the GSI-I-reduced osteogenesis

**Fig 8:**
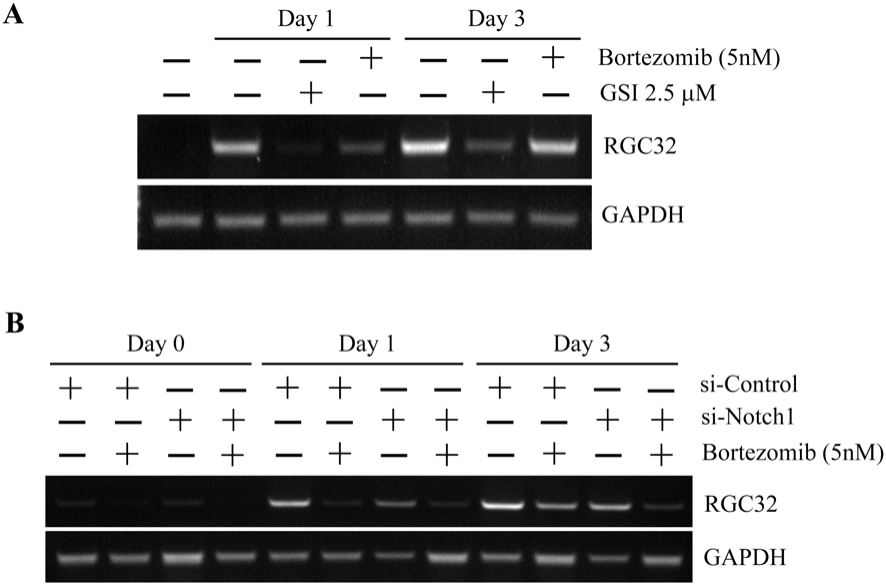
Both Notch signaling and proteasome pathway contributes to the osteogenesis of hUC-MSCs. The semi-quantitative RT-PCR shows that the treatment with 2.5 μM GSI-I significantly reduces RGC32 transcription at day 1 and day 3 after osteogenic induction (A). However, whereas 5 nM Bortezomib alone reduces RGC32 transcription at day 3, not day 1, of osteogenic induction, and siNotch transfection alone slightly reduces RGC32 transcription at both day 1 and day 3, the combination of both siNotch1 transfection and Bortezomib treatment significantly reduces the transcription at both day 1 and day 3 after osteogenic induction (B).

To next determine the contribution of Notch inhibition and proteasome inhibition to the GSI-I-inhibited osteogenesis, we then treated hUC-MSCs with siNotch1 and Bortezomib before detecting RGC32 transcription. It was found that, comparing with 2.5 μM GSI-I, which dramatically reduced RGC32 transcription at both day 1 and 3, Bortezomib alone or siNotch alone only induced the reduction at day 1, but not day 3, of the induced differentiation (Fig. 8A & B). However, siNotch1 and Bortezomib together resulted in a significant inhibition of RGC32 transcription at both day 1 and day 3 (Fig. 8B), thus strongly suggesting that both Notch1 inhibition and proteasome inhibition contributed to the GSI-I-induced reduction of osteogenic differentiation of hUC-MSCs.

### 8. The Notch1 inhibition, but not proteasome inhibition, contributed to the GSI-I-reduced immunomodulation through promoting IDO1 transcription

To also distinguish between Notch inhibition and proteasome inhibition for the contribution to the GSI-I-induced reduction of IDO1 expression and Th1 lymphocyte proliferation, we employed the same experimental design as described above by treating hUC-MSCs with both Notch1 siRNA and Bortezomb. It was found that, while Bortezomib alone had no effect on IFN-γ-induced IDO1 expression and siNotch1 transfection alone reduced over 50% of the expression, the combinatorial treatment did not further enhance the reduction of IDO1 expression (Fig. 7A, 7B and 7C). Consistently, it was found that siNotch1 alone significantly inhibited the hUC-MSC-mediated suppression of Th1 proliferation and Bortezomib alone showed no such effect, but the addition of Bortezomib treatment did not further increase this effect (Fig. 7D), thus strongly suggesting that it was Notch inhibition, but not proteasome inhibition, that contributed to the GSI-I-reduced immunomodulation of hUC-MSCs.

**Fig 9:**
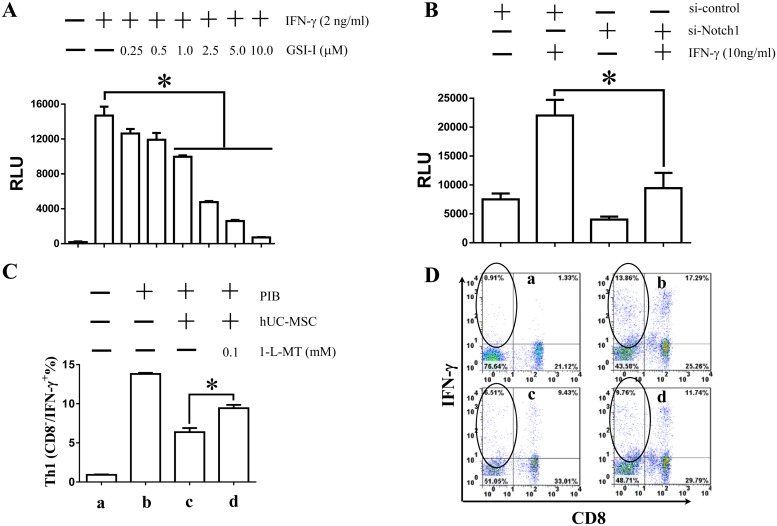
The Notch1 signaling is involved in immunomodulation of hUC-MSCs through promoting IDO1 transcription. A. Luciferase assay shows that, after transfection with pIDO1-Luc, the IDO1 promoter can be significantly activated in hUC-MSCs by 2 ng/mL IFN-γ. However, such activity can be significantly inhibited by GSI-I in a dose-dependent manner. B. Luciferase assay shows that siNotch1 transfection reduces over 50% of the IDO1 promoter activity. The * indicates statistical significance with *p*<0.05. C. Flow cytometry assay shows that treatment with 0.1 mM 1-L-MT during co-culturing of hUC-MSC and PBMCs can significantly reduce the effect of hUC-MSCs on inhibition of Th1 lymphocyte proliferation. The * indicates statistical significance with *p*<*0.05*D. D. The flow cytometry blot data are corresponding to the results of Fig. 9C. The Th1 lymphocytes circled in the plot are the CD8^-^/IFN-γ^+^ lymphocytes.

To investigate the relationship between Notch signaling and IDO1 expression in the regulation of the immunomodulation, we generated a pIDO1-Luc construct following a previous report to test the effect of Notch1 inhibition on IDO1 promoter activity [33]. It was found that, whereas the luciferase activity representing IDO1 promoter activity in the pIDO1-Luc-transfected cells was significantly increased by 2 ng/mL IFN-γ (Fig. 9A), the co-treatment with 0.1 to 10.0 μM GSI-I inhibited the IFN-γ-induced IDO1 promoter activity in a dose-dependent manner (Fig. 9A). Next, it was observed that siNotch1 reduced over 50% of the IDO1 promoter activity, thus revealing that the Notch signaling can promote IDO1 transcription in hUC-MSCs (Fig. 9B). Moreover, it was found that 0.1 mM 1-L-MT, an IDO1 specific inhibitor, significantly reduced the hUC-MSCs-mediated inhibition of Th1 lymphocyte proliferation (Fig. 9C), thus supporting a new hypothesis that the Notch signaling regulates immunomodulation of MSCs through promoting IDO1 transcription.

## Discussion

The present study reveals several novel findings all indicating that the Notch signaling may represent a critical cell signaling for maintaining key quality-related properties of MSCs, which are expression of surface markers, differentiation potency and immunomodulation. Although the Notch signaling has been reported before for its involvement in regulating osteogenic differentiation and viability of normal and tumor stem cells, respectively, its involvement in regulating surface markers and immunomodulation of MSCs has been rarely studied [37,38]. All the findings were achieved by initially employing GSI-I, a small γ-secretase inhibitor possessing inhibitory activity on both Notch signaling and proteasome activities [25,39]. This approach not only advances our understanding of the Notch signaling in MSCs, but also leads to new hypotheses that other protein(s) or pathways regulated by the ubiquitin-proteasome pathway may modify the Notch signaling activities in MSCs.

### GSI-I inhibits both Notch signaling and ubiquitin-proteasome activities in hUC-MSCs

Due to the ability to block γ-secretase-mediated intramembrane proteolysis of Notch proteins, various GSIs have been used as experimental tools in identifying novel functions of Notch signaling in different cell types [25,39]. In addition, they have also been intensely investigated in preclinical and clinical studies as potentially novel therapeutic agents for treating human cancers, which commonly exhibit abnormal Notch activities or features of cancer stem cells, such as the CD133-positive glioblastoma [40]. The therapeutic effects of GSIs lie in their abilities to inhibit tumor cell growth, induce differentiation and apoptosis, or sensitize tumor cells to radiation- or chemotherapy-induced apoptosis [41].

Gamma-secretase is a protein complex comprising of four subunits, i.e. Presenilin, Nicastrin, Aph-1 and Pen-2, and is responsible for intramembrane proteolysis of Notch as well as more than 100 other substrates [42]. Among the frequently used GSIs are leupeptin analogues, like GSI-I, sharing both structural and functional similarities with oligopeptide-type proteasome inhibitors, such as Bortezomib, an experimentally and clinically used proteasome inhibitor [25,34,43]. Therefore, given the features of multi-substrates and proteasome inhibition, extreme care should be taken when interpreting new findings derived from the GSI-based studies [44].

This study clearly demonstrates that GSI-I can elicit inhibition of Notch signaling in hUC-MSCs as both high-dose and low-dose GSI-I can reduce Notch1 cleavage (Figs. 1A & 5B-1) and low-dose GSI-I can reduce the expression of Hes1, a well-established downstream effector of the Notch signaling (Fig. 5B-1). In addition, GSI-I can also induce proteasome inhibition as shown by the increased ubiquitination of Mcl-1, a well-established degradation target of the ubiquitin-proteasome pathway (Figs. 1C & 5A). Therefore, it is likely that any effect of GSI-I on hUC-MSCs may be contributed by Notch inhibition, proteasome inhibition, or the combination of both Notch and proteasome inhibition.

### The GSI-I-induced apoptosis is attributable neither to Notch and nor to proteasome inhibition

The present study demonstrates a new finding that GSI-I can induce caspase-dependent apoptosis in hUC-MSCs as evidenced by the reduced cell viability, increased protein cleavages and DNA damage, even though the apoptosis is subjected to the regulation of multiple anti-apoptotic mechanisms. Both GSI-I and Bortezomib have been reported to induce apoptosis in cancer cells, but not yet in MSCs [31,34,40,43]. Given that siNotch1 alone, Bortezomib alone, or even the combination of both treatments fails to induce apoptosis in hUC-MSCs (Fig. 8), it is likely that a new mechanism beyond Notch and proteasome signaling may be responsible for the GSI-I-induced apoptosis in MSCs. Since γ-secretase has more than100 substrates [42], it is possible that the γ-secretase substrate(s) other than Notch family proteins may determine viability more specifically in MSCs than in other cells. Nevertheless, identification of candidate γ-secretase substrate(s) more specifically responsible for the viability of MSCs is warranted.

### Multiple mechanisms are attributable to the GSI-I-reduced CD105 expression

Another new finding revealed in this study shows that GSI-I can reduce expression of surface markers CD73, CD90 and CD105, CD105 in particular. Interestingly, the reduction in CD105, but not CD73 and CD90, can be induced additively by Notch and proteasome inhibition. However, the reduction of CD105 cannot reach the level induced by greater than 2.5 μM GSI-I, the doses of inducing apoptosis, thus suggesting the surface markers served as a part of minimal criteria for defining MSCs may be subjective to Notch, proteasome and even cell death-associated regulations. CD105 is also known as endoglin and involved in multiple functions of MSCs, such as differentiation, angiogenesis, and regenerative potential [45–47]. The more significant change observed in CD105 than in CD73 and CD90 may suggest that CD105 is more sensitive than other positive markers to the viability-associated events, thus suggesting that CD105 may be used as a surrogate marker to indicate the occurrence of cell death. In addition to the change of CD105, the combination of siNotch1 and Bortezomib treatment also reduced the positivity of CD90, which was although not as significant as the change of CD105 (Fig. 6C). Since the reduction of CD90 expression has been reported to be associated with the reduced osteogenic differentiation [9]，it is also interesting in future studies to further investigate the relationship between the CD90 expression and MSC osteogenic potential, especially in the association with the interaction between Notch signaling and the protein(s) regulated by the proteasome.

### Possible interaction between Notch signaling and the proteasome-targeted protein(s) in the regulation of osteogenic differentiation of MSCs

The Notch signaling has been reported to promote osteogenic differentiation of human MSCs as Jagged1 or NICD can promote osteogenic differentiation of hBM-MSCs [48]. In this study, given that only can the combination of siNotch1 and Bortezomib consistently inhibit transcription of RGC32, a gene well correlated with osteogenic differentiation of hUC-MSCs (Figs. 3 and 7), other protein(s) targeted by the ubiquitin-protesome system may exist to interact with Notch signaling in regulating osteogenesis of MSCs. To date, there is no evidence suggesting a direct involvement of RGC32 in regulating osteogenesis. However, one study showed that the RGC32 expression may be up-regulated by Runx2 [49], a key transcription factor that integrates functions of various cell signaling, including the Notch signaling, during osteogenesis [50]. However, surprisingly, in addition to RGC32, we have tested expression of BGLAP (or osteocalcin)，Collagen I and RUNX2, all of which were associated with osteogenic differentiation of MSCs [51], but found that the change of RGC32 gene expression was the most significant and consistent one among all genes tested (data not shown). It is thus of great interest to identify the proteasome-targeted proteins that interact with Notch signaling in the regulation of osteogenesis of MSCs using RGC32 as a useful marker.

### The Notch signaling may regulate immunomodulatory function of MSCs through regulating IDO1 transcription

The increasing evidence support that the immunomodulatory property is more responsible than the progenitor property for the efficacy of MSC-based therapies [52]. Immunomodulation is achieved through direct interaction between MSCs and various immune cells within inflammatory microenvironment and release of different immunomodulatory molecules, such as PGE2, IL10 and IDO1, from MSCs upon activation by proinflammatory cytokines, like IFN-γ and TNF-α in the environment [53]. Therefore, a cell signaling that can directly link the cell-cell interaction and release of immunomodulatory molecules will serve a much more efficient regulation than two separate cell signaling. Given our novel finding that the Notch signaling promotes IDO1 transcription, it is believed that the Notch signaling may represent such an efficient cell signaling that combines direct interaction between MSCs and adjacent immune cells through Notch receptors and the release of IDO1 directly through NICD or indirectly through downstream effector(s) of the Notch signaling.

In summary, the present study reveals several novel findings regarding regulations of surface markers, differentiation potential and immunomodulation, all of which are key quality-associated properties of MSCs. The findings indicate that the Notch signaling is likely the centerpiece in the relationship among the quality-associated properties of MSCs. More specifically, the study proposes that the Notch signaling may play a critical role in mediating the IDO1-related immunomodulation of MSCs. In addition, the study warrants identification of new molecules or signaling that interact with or modify the Notch signaling in regulating CD105 and osteogenic differentiation, or is the effectors that mediate IDO1 transcription downstream of the Notch signaling. It is believed that all these new studies will help eventually develop the cell signaling-specific quality assessment technologies for more accurately evaluating the key quality attributes of the MSC-based therapeutic products.
